# Juvenile Idiopathic Arthritis in the Era of International Cooperation

**DOI:** 10.5041/RMMJ.10278

**Published:** 2017-01-30

**Authors:** Yosef Uziel

**Affiliations:** 1 Pediatric Rheumatology Unit, Department of Pediatrics, Meir Medical Center, Kfar Saba, Israel; 2 Sackler Faculty of Medicine, Tel Aviv University, Ramat Aviv, Israel

**Keywords:** Chronic disease, education, evidence-based medicine, international cooperation, juvenile idiopathic arthritis, research

## Abstract

Juvenile idiopathic arthritis (JIA) is the most common chronic disease of childhood. Improved understanding of its pathogenesis has led to international cooperation in clinical studies. Multicenter, international collaborations and research facilitate rapid enrollment of enough patients to enable a variety of studies, including those of epidemiology, diagnostic and classification criteria, genetic disease predisposition, pathogenesis, outcomes, and treatment protocols. In the last 20 years, the vision of the Pediatric Rheumatology International Trial Organization (PRINTO) has become a reality of worldwide collaboration in pediatric rheumatology research, including North American and European research groups. Major advances have been made in treating systemic JIA and its main complication, macrophage-activating syndrome (MAS). Single Hub and Access Point to Pediatric Rheumatology in Europe (SHARE) is a project of the European Society of Pediatric Rheumatology with the goal of improving clinical care. Based on evidence in the scientific literature, position papers regarding optimal clinical approaches and care have been published. Formal, validated assessment tools to evaluate response to treatment have been developed. Recommendations have been established to encourage international research collaborations, especially in light of major advances achieved in the genetics of pediatric rheumatologic diseases and the need to share biological samples among different countries and continents. Every participating country has disease information available for patients and families. Additionally, educational programs and updated syllabi for pediatric rheumatology have been written to promote similar, high-level academic training in different countries. These efforts have resulted in significant improvements in treatment and in patient prognosis. However, improved cooperation is needed to enhance research with biological and genetic samples. The Israeli Research Group for Pediatric Rheumatology is very active and has made significant contributions to the field.

## INTRODUCTION

One in 1,000 children is affected by the various subtypes of chronic juvenile idiopathic arthritis (JIA).^1^ The field of pediatric rheumatology exemplifies a voluntary international cooperative effort. This cooperation facilitates multicenter studies that can enroll hundreds of patients in a relatively short period. These research efforts promote better understanding of disease progression and help develop adequate treatment protocols. Many centers in Israel are active participants in these studies.

Cooperative studies have been conducted in many areas of pediatric rheumatology, including systemic lupus erythematosus, juvenile dermatomyositis, vasculitis, and autoinflammatory diseases, to name a few. In this review we will focus on three recent, major targets for cooperation: JIA therapy, systemic onset JIA (SoJIA), and macrophage-activating syndrome (MAS).

Due to cooperative efforts over the last few decades, the mechanisms underlying these diseases have been progressively elucidated, and as a result treatment has improved tremendously. The once frequent image of a sick child sitting in a wheelchair is nowadays very rarely seen. The goal of medical care today, the “treat-to-target” (T2T) approach, is disease remission, or at least to achieve low disease activity with minimal side effects. Most children today can achieve this goal.^2^

## COOPERATIVE EFFORTS AND ORGANIZATIONS

Pediatric rheumatologic diseases are relatively rare. Some might even be considered orphan diseases. Thus, cooperative efforts have been undertaken in several areas. One objective was to obtain sufficient patient populations for clinical therapeutic trials. Another goal was to standardize assessment of disease activity, to enable every center to use the same internationally accepted and validated assessment tools. Improved cooperation is also needed among patient and parent organizations, with the vision of improving the care of pediatric rheumatology patients, globally.

Joint collaborative research in pediatric rheumatology has been conducted successfully for several years. The main study groups are The Childhood Arthritis & Rheumatology Research Alliance (CARRA), Pediatric Rheumatology Collaborative Study Group (PRCSG), and the Pediatric Rheumatology International Trial Organization (PRINTO). The American College of Rheumatology (ACR), European League Against Rheumatism (EULAR), and the Pediatric Rheumatology European Society (PRES) are considered the primary rheumatology societies supporting cooperative efforts. A few examples resulting from this international cooperation, mainly between PRINTO and CARRA-PRCSG, can be found in the literature.^3^–^12^ Some focused on finding improved medications,^3^–^8^ and MAS was the focus of other studies.^9^–^12^

Advances have occurred primarily under the auspices of PRINTO.^13^ The ultimate goal of PRINTO is to encourage and support knowledge acquisition and research in pediatric rheumatology. The PRINTO group was established in 1996 by Nicola Ruperto and Alberto Martini from Italy. It is now celebrating 20 years of achievements and success. As of January 2017, PRINTO includes 1,735 members in 596 medical centers, in 81 countries (64 with a National Coordinator).

Another more recent major cooperative effort is the SHARE project, which stands for Single Hub and Access Point to Pediatric Rheumatology in Europe. The SHARE project was established in 2012, funded by the European Union. It was developed with the express goal of optimizing cooperation among rheumatology scientists and physicians.^14^ The specific aims of the projects are to improve the quality of treatment in rheumatologic diseases by using a patient- and family-centered approach, to provide optimal treatment based on current literature, to promote international research, to improve patient access to information regarding diseases and medical centers, and to enhance specialty education in each country.

One of the aims of the SHARE initiative was to map the current state of the disease in every country in Europe regarding pediatric rheumatology centers, medical and paramedical staff, patient access, treatment with advanced biological medications, accessibility of joint injections, etc. Internet questionnaire surveys were conducted in each country. National representatives of PRINTO, pediatric rheumatology centers, representatives of parent societies for childhood arthritis, and parents of children with JIA participated.

As expected, accessibility and level of treatment are better in Western than in Eastern European countries. Remarkably, Israel is one of the leading countries in the field in terms of the level of medical treatment, use of novel biological drugs, and very good accessibility to pediatric rheumatologists.

## TREATMENT GOALS AND ADVANCES

The main goals of treatment are preventing joint damage, ameliorating systemic signs, improving quality of life of patients and parents, preventing bone loss, preventing growth retardation, and enabling the child and family to participate in a healthy social life and in educational and peer activities.

Advances in treatment have been achieved due to increased understanding of the molecular mechanisms involved in JIA. This includes the role of the cytokines underlying the inflammation, which enables blocking their activity. The first generation of biological medications targeting the cytokines signaling disease activity included anti-tumor necrosis factor (TNF) agents. These medications profoundly changed the course of the disease, while improving the health and quality of life of the sick children. Subsequently, after basic research and discovery of the roles of other cytokines in inflammation, other rationalized biological drugs were developed. These include those that block other cytokines, such as interleukin (IL)-1 or IL6 (the pathogenic cytokines in SoJIA), abatacept, which targets the connections between antigen-presenting cells and T cells, and rituximab, which blocks B cells.

### Systemic Onset JIA

One of the most vicious diseases in the field of pediatric rheumatology is systemic onset JIA (SoJIA). Systemic onset JIA is characterized by high, spiking fever once or twice a day, rash, hepatosplenomegaly, lymphadenopathy, pericarditis, and very high, inflammation-related laboratory parameters due to high pro-inflammatory cytokine levels. Most patients develop severe chronic disease with a strong dependence on steroids, failure to grow, and destructive arthritis. Their quality of life is poor.

The mechanism underlying disease progression involves increased IL1 and IL6 cytokine activity. Drugs that target these cytokines induce disease remission and are currently the treatment of choice. Multicenter studies involved 177 patients in the canakinumab trial^7^ and 112 in the tocilizumab^8^ trial. These were conducted in the new way of studies in JIA, with a withdrawal phase following an open phase (described in detail in the Clinical Trials section). Both studies demonstrated good efficacy and safety. These novel therapies minimize side effects and avoid the need for steroids as a clinical treatment. A *New England Journal of Medicine* editorial accompanying the publication of these two phase III studies stated that they represent “a new era in the field of Pediatric Rheumatology.”^15^ Most patients today achieve remission.^2^

### Macrophage-Activating Syndrome

Among children with SoJIA, 10% might develop macrophage-activating syndrome (MAS), a severe, life-threatening complication evolving from high levels of pro-inflammatory cytokines. An international effort to clarify this syndrome led to the cooperation of many centers for data collection. It included SoJIA patients with MAS, patients with active SoJIA but without MAS, and a control group with bacterial infections. Analyzing this large data set helped define several criteria of this syndrome, as well as recommendations for treatment that were determined by a consensus committee meeting of senior pediatric rheumatologists and hematologists.^9^,^10^,^12^,^16^

## CLINICAL TRIALS

In the past, randomized, placebo-controlled trials were conducted to assess the effects and side effects of new therapies. Today, a more ethical practice is followed for clinical studies in pediatric rheumatology. In the first stage, all patients are treated with the active drug. In the second stage, patients that responded according to the accepted criteria are divided into treatment and control groups, and the time until relapse in all patients is followed. In the third stage, all patients in both groups are again treated with the active medication, including those that relapsed during the second stage of the study while on placebo. Patients who did not respond to the drug in the first stage may be treated with it again in the third stage.

Conducting such studies in JIA demonstrated the efficiency and safety of anti-TNF compounds such as etanercept,^17^ infliximab,^3^,^4^ adalimumab,^5^ and abatacept (a specific inhibitor of the co-stimulator of T cells that bind to the binding sites of the antigen-presenting cells, thus inhibiting cell activation and inflammation processes).^6^ Following these studies, which demonstrated efficacy and safety, the drugs were registered as optional therapies for JIA. Table 1 summarizes multicenter international PRINTO and PRCSG trials of biologic drugs.

**Table 1:** Multicenter International PRINTO and PRCSG Trials of Biologic Drugs for JIA.

Study, Ref Number	Medication	Patients (*n*)	Indication	Israeli Usage Protocol
Lovell et al.^17^	TNFα inhibitor—etanercept	69	JIA	Second line after MTX failure
Ruperto et al.^3^	TNFα inhibitor—infliximab	122	JIA	Second line after MTX failure
Lovell et al.^5^	TNFα inhibitor—adalimumab	171	JIA	Second line after MTX failure
Ruperto et al.^6^	Co-stimulator T cell stimulation inhibitor—abatacept	190	JIA	Second line after MTX failure and/or anti-TNF failure together with MTX
Ruperto et al.^7^	Anti-IL1—canakinumab	177	SoJIA	Third line after CS and tocilizumab failure
De Benedetti et al.^8^	Anti-IL6—tocilizumab	112	SoJIA	Second line after CS failure

CS, corticosteroids; IL1, interleukin 1; IL6, interleukin 6; JIA, juvenile idiopathic arthritis; MTX, methotrexate; SoJIA, systemic onset juvenile idiopathic arthritis; TNF, tumor necrosis factor.

## DISEASE ASSESSMENT TOOLS FOR TREATMENT RESPONSE

Historically, response to therapy was evaluated subjectively by the treating physician. Formal validated assessment tools are used today. The American College of Rheumatology (ACR) established six criteria to evaluate response to treatment. These include a general assessment and physical examination by a physician, and patient or parental evaluation including subjective assessment of the general disease (a functional questionnaire about physical and psychological daily function such as, for example, the Child Health Assessment Questionnaire (CHAQ), physical examination counting the number of active inflamed joints, and one laboratory parameter of inflammation). The ACR response criteria are evaluated as follows: clinical response is considered good when at least 30% improvement in at least three of the six parameters is achieved. One criterion might deteriorate and become worse. The functional questionnaire was validated in every participating country, including Israel.^18^

In recent years, the Juvenile Arthritis Disease Activity Score (JADAS) has become widely used, among other reasons due to its user-friendly structure.^19^,^20^ The JADAS is composed of three or four variables (including or excluding the parameter on inflammation). These include: (1) Physician’s global assessment of disease activity measured on a 0–10 visual analog scale (VAS), where 0=no activity and 10=maximum activity; (2) Parent or patient global assessment of well-being measured on a 0–10 VAS, where 0=very well and 10=very poor; (3) count of joints with active disease assessed in 71, 27, or 10 joints (JADAS71, JADAS27, and JADAS10, respectively); and (4) The erythrocyte sedimentation rate (ESR) normalized to a 0-to-10 scale. The JADAS is calculated as the sum of these scores.

This evaluation score was also validated in the language of every participating country, including in Hebrew. According to the results of this assessment, disease activity is defined as in remission, mild, moderate-to-severe, or relapsed. When the patient is categorized with moderate-to-severe disease activity, enhanced treatment should be considered.

### Developing Clinical Guidelines

One important goal of SHARE was for the European Union for Pediatric Rheumatology to establish and implement guidelines for diagnosing and treating rheumatologic diseases of childhood, in parallel to the guidelines written for adult rheumatology patients.^21^ Leading senior specialists, pediatric rheumatology fellows, and a librarian that was responsible for data-mining according to key words using MESH terms participated in the process.

A thorough search of the literature from 1970 to the present was performed using PubMed, Embase, and Cochrane databases. The most relevant papers were chosen by the primary researchers. The strength and validity of each study was evaluated by at least two independent specialists. Based on the information obtained from the chosen papers, recommendations for diagnosis and treatment were framed. These were validated by a wider group of specialists.

A consensus meeting was held, and the final recommendations were established using the Delphi technique. An 80% majority was required to approve an item. In this way, updated, evidence-based recommendations for diagnosing and treating JIA were established.^22^

### Education for Patients and Parents

The content of the PRINTO website (www.printo.it/pediatric-rheumatology) was written by an international group of senior pediatric rheumatologists. It provides professional, reliable, up-to-date information regarding all the pediatric rheumatology diseases and treatments, for parents of patients with these diseases. The information is available in almost 50 languages. In Israel there are over 1,000 new entries to the website each month, highlighting the relevance and importance of this site.^23^

A comic book was written for sick children. It tells a story that clarifies the mechanisms of JIA and its various treatments. A recent Israeli study found that reading this booklet increased short- and long-term knowledge among patients.^24^ Patients’ knowledge at baseline was 62% and rose to 80% after reading the comic book. This knowledge was maintained after a year.

### Medical Education and Training

A unified syllabus for fellowship training in Europe was developed for pediatric rheumatology and was validated by the European Health Authority. Fellowship programs in several medical centers in various countries are included. The goal is to increase the number of fellowships and include the knowledge discovered by specialists in pediatric rheumatology and other scientific groups. This will contribute to promoting research and increasing the quality of treatment while decreasing costs due to synergistic effects based on optimization of treatment.

In Europe, 42% of the countries acknowledge pediatric rheumatology as a sub-specialty. In Israel, fellowship training in pediatric rheumatology is acknowledged by the Scientific Council. Several pediatric rheumatology centers have been accredited for this purpose.

### Improving International Research Efforts

Although current cooperative efforts are progressing, improvements are needed because current areas of research include new, wide-ranging fields such as biology, genetics, and DNA sample collection, among others. It is important to alleviate the barriers and limitations to cooperation among countries and to increase accessibility to biological and genetic data. This is true from the aspects of protocol approval, data collection, and biological bio-banking sample collection for genetic studies. One of the goals of the SHARE project was to establish a combined Helsinki authority for all countries to help fulfill these goals.^25^ The aims are to remove barriers between established networks and international projects, between individuals’ diseases and medical staff, and to promote friendly access to drug development from the early concept stages through advanced clinical studies. Progress in this area is lagging, however.

## ISRAELI STUDIES AND NATIONAL AND INTERNATIONAL COOPERATION

On a national level, the Israeli Research Group for Pediatric Rheumatology includes all Pediatric Rheumatology centers in the country. An advantage of a small country such as ours is that specialists can lead a study in their sub-specialty, while maintaining high academic and clinical standards. Our national research group has contributed a great deal to conducting and publishing several studies in pediatric rheumatology that have received international acknowledgment in JIA research.^26^–^29^ These include a study on the epidemiology and prevalence of pediatric rheumatological diseases in Israel,^26^ another that did not find seasonality in SoJIA and did not confirm the hypothesis of possible viral triggers of disease onset,^27^ the finding of a high prevalence of antithyroid antibodies in a larger series of JIA,^28^ and a new report on methods of nitrous oxide sedation for intra-articular injections for JIA.^29^

## SUMMARY

Due to current treatment advances, most JIA patients will enter a remission state of their disease. Likewise, SoJIA, which is considered one of the most severe disease subtypes, is now treatable, with the majority of affected children achieving remission.

The field of pediatric rheumatology is a model for international medical collaboration. Due to this cooperation, evidence-based medicine guidelines for diagnosis and treatment have been established, which promote good health and quality of life for patients. The SHARE project was able to achieve all its goals within three years. These included agreement on minimum standards of care in every country, based on the best known therapies for JIA; developing established recommendations for diagnosis and treatment of the diseases in the field; providing easy access to information for parents of sick children; and promoting basic and clinical research and teaching. The hope is that other countries (particularly in Eastern Europe) will benefit in these areas, which will promote improvements in diagnosis, treatment, research, and education. Importantly, Israel is one of the leading countries in patient accessibility to pediatric rheumatologists and in having high standards of care. This is definitely due to the devotion of the pediatric rheumatologists in the country.

## Abbreviations

JADAS: juvenile arthritis disease activity score
JIA: juvenile idiopathic arthritis
MAS: macrophage-activating syndrome
PRINTO: Pediatric Rheumatology International Trial Organization
SHARE: Single Hub and Access Point to Pediatric Rheumatology in Europe
SoJIA: systemic onset juvenile idiopathic arthritis
